# Neuronal Primary Cilia Mediate Noggin Release to Enable Extracellular Signaling

**DOI:** 10.3390/cells14201607

**Published:** 2025-10-16

**Authors:** Sara R. Dunlop, Justin A. Geier, Chian-Yu Peng, John A. Kessler

**Affiliations:** Department of Neurology, Northwestern University Feinberg School of Medicine, Chicago, IL 60611, USA; dunlop.sara@mayo.edu (S.R.D.); cypeng@northwestern.edu (C.-Y.P.); jakessler@northwestern.edu (J.A.K.)

**Keywords:** primary cilia, noggin, BMP, sonic hedgehog, neurogenesis

## Abstract

The primary cilium is generally viewed as a sensory organelle that transduces chemical and mechanical stimuli from the environment. In the adult hippocampus, primary cilia mediate the effects of sonic hedgehog (Shh) and other signals on neurogenesis and hippocampal function, and loss of cilia leads to cognitive and behavioral deficits. The secreted peptide noggin is a bone morphogenetic protein (BMP) antagonist and plays a critical role in regulating adult hippocampal neurogenesis (AHN) and hippocampus-dependent behavior. Here, we show that noggin is expressed by mature granule cell neurons, that it is apically targeted and localized intracellularly near the pocket region of primary cilia, and that cilia regulate noggin release through Shh and somatostatin (SST) pathways. Further, granule cell activation modulates noggin dynamics both in vitro and in vivo. Together, these findings demonstrate synergy between Shh and noggin and the positive regulatory action of neuronal activity on regulating BMP antagonism within the neurogenic niche. Thus, the primary cilium is not only an organelle that transduces signals to neurons but also one that mediates extracellular signaling. Significance statement: Primary cilia are organelles that protrude from the surface of most vertebrate cell types. Defects in primary ciliary structure and function are associated with human disease. Primary cilia are generally viewed as exclusively sensory organelles that respond to environmental signals to regulate both cell development and adult cell function. This study demonstrates that the primary cilia in hippocampal granule cell neurons mediate the release of the BMP antagonist, noggin. These observations expand the current understanding of ciliary signaling and may inform future studies exploring the connection between hippocampal activity and cognition in ciliopathies.

## 1. Introduction

Hippocampal neurogenesis, defined by the generation of new granule cell neurons in the dentate gyrus (DG), continues throughout life in both animals and humans but declines with chronological aging [1,2,3,4]. Altered neurogenesis is associated with changes in both cognitive and affective behavior in animal models [5,6,7,8]. Exposure to exercise or to environmental enrichment increases neurogenesis and can enhance adaptive abilities to partially restore cognitive and coping functions that are susceptible to age-related decline [1,9,10,11,12]. While exercise and environmental enrichment increase immediate early gene (IEG) expression, a proxy of cellular activity, the molecular mechanisms by which DG neurons respond to activity to modulate adult hippocampal neurogenesis are not fully understood.

Neurogenesis is tightly regulated through multiple signaling pathways, such as Wnt, Notch, sonic hedgehog (Shh), brain derived neurotrophic factor (BDNF), and bone morphogenic proteins (BMPs) [13,14,15,16,17,18]. While Shh promotes adult neurogenesis, BMP serves as a negative regulating factor for the birth of new neurons [18]. Noggin is the predominant BMP inhibitor in postnatal and adult hippocampi and functions to increase neurogenesis by inhibiting the binding of extracellular BMPs to the membrane-bound BMP receptor [3,19,20,21,22]. In the DG, noggin is expressed by granule cells [23,24,25,26], but the mechanisms regulating noggin release and how it ultimately affects the neurogenic niche remain incompletely understood.

In the nervous system, primary cilia are solitary organelles present on the apical surface of both neurons and glia. These non-motile structures sense and respond to both chemical and physical stimuli and are enriched for GPCR expression [27]. Primary cilia are necessary for Shh signaling, representing the site of the Shh receptor, Patched, and the signaling molecule smoothened (SMO), which translocalizes to the nucleus after Shh binds to Patched [28]. Furthermore, primary cilia are required to mediate the stimulatory effects of Shh signaling on hippocampal neurogenesis [28,29]. Of the many GPCRs found on cilia and throughout the cell, somatostatin receptor 3 (SSTR3) is unique in that it is exclusively localized to primary cilia and has been implicated in modulating excitatory synaptic inputs [30].

The present study examined factors regulating noggin expression and release in the DG. We found that noggin is targeted to the apical surface of DG granule cells, where it associates with primary cilia, and noggin expression and release are regulated through ciliary GPCR signaling. We observed that Shh signaling and neuronal depolarization each increase extracellular release of noggin. By contrast, SST signaling through the SSTR3 receptor found exclusively on cilia produced the opposite effects and led to reduced release and intracellular retention of noggin. Taken together, these findings expand current concepts about the functions of primary cilia by demonstrating a role in extracellular signaling and may assist in understanding the clinical heterogeneity of human ciliopathies and neurodegenerative disorders.

## 2. Materials and Methods

### 2.1. Ethics Statement

All animal procedures were approved by the Northwestern University Institutional Animal Care Use Committee (IACUC) review board. All experiments were performed in accordance with the Public Health Service Policy on Humane Care and Use of Laboratory Animals. Northwestern University has an Animal Welfare Assurance on file with the Office of Laboratory Animal Welfare (A3283-01). Northwestern University conducts its reviews in accordance with United States Public Health Service (USPHS) regulations and applicable federal and local laws. The composition of the Northwestern University IACUC meets the requirements of USPHS policies and Animal Welfare Act regulations updated 1 April 2025.

### 2.2. Mice

All animals were housed in standard animal housing, with temperature and humidity controlled, under a 14:10 light/dark cycle, with access to food and water ad libitum. Animals were housed 2–5 per cage. Behavioral testing was conducted during the light cycle. C57Bl/6 wild-type animals were used as controls (The Jackson Laboratory, Bar Harbor, ME, USA). IFT88^flx/flx^ colonies were generated from homozygous breeding pairs (Jackson Laboratory stock #022409). Calb1-IRES2-Cre-D knockin mouse colonies were generated from homozygous breeding pairs (Jackson Laboratory stock #028532). Homozygocity was confirmed through genotyping PCR (Transnetyx, Inc., Cordova, TN, USA) initially upon animal receipt and periodically thereafter to ensure genotype of progeny. IFT88^flx/flx^ animals were used at 6–8 months of age for all in vivo experiments. Animals were randomly assigned to running or sedentary groups. Equal numbers of male and female animals were used in behavioral experiments.

### 2.3. Small Interfering RNAs

siRNAs (Thermo Fisher Scientific, Waltham, MA, USA), (Santa Cruz Biotechnology, Inc., Dallas, TX, USA) against either noggin, RAB8a, or SSTR3 were delivered in vitro using the Lipofectamine 3000 system (Thermo Fisher Scientific, Waltham, MA, USA) and used according to the manufacturer’s provided protocol. Cells were treated with siRNA constructs or vehicle for 6 h before Lipofectamine was removed, washed with phosphate buffer saline (PBS), and fed with fresh media containing supplements. Subsequent analysis or manipulation was performed 24–72 h after Lipofectamine treatment to ensure adequate knockdown of the protein target. All knockdown experiments were confirmed and validated through western blots to ensure target protein reduction.

### 2.4. Drug Administration

#### 2.4.1. Potassium Chloride (KCl)

KCl was prepared and mixed under gentle agitation in DMEM F12 media (Thermo Fisher Scientific, Waltham, MA, USA) to create a working dilution of 100 mM KCl. KCl was prepared fresh prior to each experiment. Cell cultures were treated at a final concentration of 20 mM KCl diluted in DMEM F12 media with necessary supplements and growth factors. Vehicle controls were treated with DMEM F12 media alone.

#### 2.4.2. Sonic Hedgehog (Shh)

Shh (MilliporeSigma, Burlington, MA, USA, Cat# GF174) was prepared from lyophilized stock in PBS and used at a final concentration of 0.01 µg/mL supplemented into prepared media. PBS was used as a vehicle control.

#### 2.4.3. Somatostatin (SST)

SST (Thermo Fisher Scientific, Waltham, MA, USA, Cat# AAJ66274MCR) was prepared from lyophilized stock in PBS and used at a final concentration of 0.01 µM supplemented into prepared media. PBS was used as a vehicle control.

#### 2.4.4. PKA Inhibitor (H89)

The PKA inhibitor, H89 dihydrochloride (Tocris Bioscience, Bristol, UK, Cat. No. 2910) solid, was dissolved in PBS at a working concentration of 100 µM and used at a final concentration of 20 µM in prepared media.

#### 2.4.5. Smoothened Agonist (SAG)

Smoothened Agonist (SAG) (Selleck Chemicals LLC, Houston, TX, USA) was dissolved in dimethyl sulfoxide (DMSO) and administered intraperitoneally once daily for 5 days at 20 mg/kg. The final volume ranged between 200 and 400 μL per animal. Control animals received equivalent volumes of 5% DMSO in saline intraperitoneally.

#### 2.4.6. Cytosine Arabinoside (AraC)

Experiments performed using AraC used a final concentration of 1 µM prepared from a 10 mM stock solution to limit glial proliferation.

### 2.5. Running/Exercise

Animals were allowed to run freely on a running wheel placed within the home cage for a duration of two weeks. Running animals were housed with 2–3 animals per cage to allow for adequate exercise per animal. After two weeks, animals were euthanized and brain tissues were collected for protein and RNA analysis.

### 2.6. Tissue Preparation

Mice were anesthetized with CO_2_ and perfused transcardially with ice-cold PBS. Brains were hemisected; the right DG was microdissected in Hanks Basic Salt Solution (HBSS) on ice and flash frozen using dry ice for future protein and RNA analysis. The left hemisphere was fixed by submersion in 4% paraformaldehyde for 24 h at 4 °C and cryoprotected with 30% sucrose for at least 72 h at 4 °C prior to sectioning. Cryoprotected brains were embedded in Tissue-Tek OCT Compound (Sakura Finetek, Torrance, CA, USA), sectioned using a cryostat (Leica Biosystems, Nussloch, Germany) at a thickness of 12 um, and sampled through the rostral caudal extent of the dentate gyrus.

### 2.7. Viral Vectors

Viral vectors were transduced in vitro and allowed to express for a minimum of 48 h prior to experimental manipulation and/or collection. Viral vectors were introduced in vivo via stereotactic injection directly into the hippocampus. In vivo, viral vectors were allowed to express for a period of two weeks prior to protein, RNA, or immunofluorescence analyses. Viral titers were used at ≥1.0 × 10^13^ vg/mL. Expression and anatomical confirmation of adequate injection was confirmed using immunofluorescent labeling with GFP.

### 2.8. AAVs

AAV viral vectors were commercially purchased from Addgene (Addgene, Watertown, MA, USA). pENN.AAV.Camkii.GFP.Cre was purchased from Addgene (Addgene, Watertown, MA, USA, ID# 105551) with an AAV9 serotype to ensure effective transduction in neuronal populations in IFT88^flx/flx^ mice. pENN.AAV.Camkii.GFP was used as a control vector for IFT88^−/−^ experiments. AAV5-hSyn-DIO-hM3Dq-mCherry and AAV5-hSyn-DIO-mCherry were purchased from Addgene (Addgene, Watertown, MA, USA, ID# 44361, 50459). Viral preps were validated in vitro prior to stereotaxic use. The viral titer was ≥7 × 10^12^ vg/mL for Addgene #44361 and 50459 and ≥1 × 10^13^ vg/mL for Addgene #105551. AAV serotype (AAV-9) was selected for targeted expression in neurons.

### 2.9. DREADDs

To selectively modulate neuronal activity in mature dentate granule cells, we employed excitatory DREADD hM3Dq, a synthetic Gq-coupled muscarinic receptor activated by clozapine-N-oxide (CNO). AAV5-hSyn-DIO-hM3Dq-mCherry and AAV5-hSyn-DIO-mCherry (Addgene: #44361, 50459) (Addgene, Watertown, MA, USA) were stereotactically injected bilaterally into the dentate gyrus of Calb1-Cre mice to achieve Cre-dependent expression in calbindin-positive granule neurons.

### 2.10. CNO Activation

CNO was purchased from Hello Bio (Hello Bio Inc., Princeton, NJ, USA) and delivered orally, suspended in drinking water. CNO was administered in drinking water ad libitum at a concentration of 0.25 mg mL^−1^ for a daily dose of approximately 5 mg/kg.

### 2.11. Stereotaxic Viral Injection

Mice were anesthetized using isoflurane and received pain management prior to survival surgery. Stereotaxic viral injections were performed using a Motorized Mouse Stereotaxic Instrument (Stoelting Co., Wood Dale, IL, USA), Prototypical Stereotaxic Injector (Stoelting Co., Wood Dale, IL, USA), and a 5 μL Hamilton micro syringe (Hamilton Company, Reno, NV, USA). A midline scalp incision was made, and craniotomies were performed at coordinates. Relative to the bregma, the coordinates were: 2 mm posterior, 1.5 mm lateral, and 1.9 mm ventral. Next, 1 µL of adeno-associated virus was injected at a rate of 0.5 µL per minute into the hippocampus. The virus was allowed to diffuse for 2 min prior to syringe removal. Control animals received a GFP-expressing adenoviral vector following the same surgery methods. Animals were subsequently sutured and monitored until conscious, alert, and active. Viral expression was subsequently confirmed through immunofluorescence after animal sacrifice.

### 2.12. Immunohistochemistry

Cryoprotected brains were embedded in OCT, sectioned using a Leica cryostat (Leica Biosystems, Nussloch, Germany) at a thickness of 12 µm, and sampled through the rostral caudal extent of the dentate gyrus. The 12 µm thick cryosections were used for immunohistochemistry. Sections were post-fixed with 4% paraformaldehyde and washed with phosphate buffered saline (PBS) (Bio-Rad Laboratories, Hercules, CA, USA). Sections were permeabilized with 1% Triton in PBS (PBST). Heat-mediated antigen retrieval was performed in pH 6.5 citrate buffer for 1 min at 95–100 °C. All washes were performed 3× with PBS. Sections were subsequently blocked in 2% bovine serum albumin (BSA) in PBST (Thermo Fisher Scientific, Waltham, MA, USA) for 1 h prior to primary antibody incubation. Secondary antibodies specific to the host of the primary antibody were incubated at room temperature and used at 1:500. Coverglasses were fixed in 4% formaldehyde solution and washed 3 times with ice-cold PBS. Fixed coverglasses were stored in PBS with 1% azide until stained. The coverglasses were washed in PBS prior to permeabilization with PBS with 1% Triton-X (PBST) for 5 min. The coverglasses were then blocked in 2% BSA prepared in 1 h at room temperature. After blocking, the coverglasses were incubated in primary antibody overnight at 4 °C. After primary antibody incubation, the coverglasses were washed 3 times with PBST for 5 min and incubated with secondary fluorescent antibodies for 1 h at room temperature. The coverglasses were mounted on slides by inverting the coverglasses in mounting media onto standard microscopy slides. The coverglasses were imaged using confocal microscopy.

### 2.13. In Situ Hybridization

Next, 20 mm thick cryosections from desired brain regions were washed 3 times with PBST and treated with Proteinase K (1 μg/mL, Thermo Fisher Scientific, Waltham, MA, USA) for 5 min. The sections were then washed 3 times in PBST and fixed in 4% paraformaldehyde and 0.1% glutaraldehyde for 30 min. Following 3 PBST washes, Digoxygenin (DIG)-labeled antisense mRNA probes (1:150 in hybridization buffer) were added to the slides, which were then covered with siliconized glass coverslips and placed in a humidified hybridization chamber at 68 °C overnight. On the following day, the slides were washed twice at 68 °C with high-stringency wash buffer for 1 h and 3 times with low-stringency wash buffer for 1.5 h. The slides were then washed 3 times with TBST followed by overnight incubation in anti-DIG alkaline phosphatase antibody Roche, 1:2000 (Roche Diagnostics GmbH, Mannheim, Germany) in 5% goat serum. The slides were subsequently washed in TBST + 2 mM levamisole 3 times and transferred into NBT-BCIP (Roche Diagnostics GmbH, Mannheim, Germany) containing developing solution from 6 h to overnight. Finally, the sections were dehydrated through an ethanol gradient, cleared in xylene, and cover-slipped with Histomount (Invitrogen, Thermo Fisher Scientific, Waltham, MA, USA) for analysis and storage.

### 2.14. Immunocytochemistry

Coverglasses were fixed in 4% formaldehyde solution and washed 3 times with ice-cold PBS. Fixed coverglasses were stored in PBS with 1% azide until stained. The coverglasses were washed in PBS prior to permeabilization with PBS with 1% Triton-X (PBST) for 5 min. The coverglasses were then blocked in 2% BSA prepared within 1 h at room temperature. After blocking, the coverglasses were incubated in primary antibody overnight at 4 °C. After primary antibody incubation, the coverglasses were washed 3 times with PBST for 5 min and incubated with secondary fluorescent antibodies for 1 h at room temperature. The coverglasses were mounted on slides by inverting the coverglasses in mounting media onto standard microscopy slides. The coverglasses were imaged using confocal microscopy.

### 2.15. Cell Culture

#### 2.15.1. Progenitor Cell Cultures

Cultures were generated from postnatal days 0–3 C57Bl/6 wild-type mice for progenitor cell culture experiments. Pups were anesthetized on ice and the bilateral hippocampi were dissected in ice-cold HBSS (Corning Incorporated, Corning, NY, USA) and placed into serum-free media. Tissue was homogenized using scissors prior to centrifugation. The resulting cell pellet was resuspended in serum-free media in the presence of 10 ng/mL EGF (MilliporeSigma, Burlington, MA, USA) and filtered prior to being placed in non-tissue culture-treated flasks. Neurosphere cultures were incubated under standard conditions at 37 °C with 5% CO_2_. Neurospheres were dissociated into single cells for progenitor cell experimental treatments.

#### 2.15.2. Neurosphere-Derived Hippocampal Neuronal Cultures

Neuronal cultures were generated from neurospheres to preserve regional specific patterning of the neurogenic niche. Neurospheres were generated from postnatal days 0–3 C57Bl/6 wild-type or IFT88^flx/flx^ mice depending on the experiment. Pups were anesthetized on ice and the bilateral hippocampi were dissected in ice-cold HBSS (Corning Incorporated, Corning, NY, USA) and placed into serum-free neurosphere propagation media, as previously described [24]. Tissue was homogenized using scissors prior to centrifugation. The resulting cell pellet was resuspended in serum-free neurosphere propagation media in the presence of 10 ng/mL EGF and 250 ng/mL noggin (R&D systems, Minneapolis, MN, USA) and filtered prior to being placed in non-tissue culture-treated flasks. Neurosphere cultures were incubated under standard conditions at 37 °C with 5% CO_2_. Neurospheres were maintained in suspension to allow the formation of 3-dimensional cultures. Neurospheres were allowed to expand for 7–10 days with media changes every 3 days. Neurospheres were then dissociated to single cells and plated at a density of 400,000 cells per well in a 12-well dish on poly-d-lysine and laminin double-coated plates or coverglasses and allowed to differentiate in the presence of 10 ng/mL BDNF (R&D systems, Minneapolis, MN, USA) and 20 ng/mL NT-3 (R&D systems, Minneapolis, MN, USA) for 28–32 days prior to experimental treatment and collection. Maturation and neuronal identity were confirmed through the expression of calbindin and beta-III-tubulin.

### 2.16. Sandwich ELISA

Conditioned cell culture media was collected from primary neuronal cultures. Conditioned media was immediately placed on ice prior to centrifugation. Media was concentrated 50–100× using Amicon centrifugation filter units (Millipore Stock #UFC801024, MilliporeSigma, Burlington, MA, USA) and centrifuged at 1200–1500 rcf for 20 min at 4 °C. Samples were stored at −80 °C until ELISA analysis (AVIVA Cat #OKEH06388, Aviva Systems Biology, San Diego, CA, USA). Samples were thawed on ice and run in duplicate or triplicate from the same differentiation experiment. Non-conditioned concentrated media was used as the control for blank wells and protein standard diluent. Measurements were calculated based on the standard curve after subtracting the average background signal. The average background was calculated by averaging the luminescent values of blank (i.e., unconditioned media) control wells.

### 2.17. Western Blot

Microdissected tissue was homogenized in T-PER (Fisher Scientific) with HALT protease inhibitor (Thermo Fisher Scientific, Waltham, MA, USA). Cell culture lysates were collected in M-PER (Fisher Scientific) with HALT protease inhibitor (Thermo Fisher Scientific, Waltham, MA, USA). All samples were centrifuged at 12,000 rpm for 15 min at 4 °C. The supernatant was then separated for protein analysis. Protein concentrations were determined using the BCA assay (Thermo Fisher Scientific, Waltham, MA, USA) and compared to bovine serum albumin (BSA) standards. Protein concentrations were measured using a NanoDrop 2000c spectrophotometer (Thermo Fisher Scientific, Waltham, MA, USA) and associated software (NanoDrop 2000/2000c software, version 1.6.198; Thermo Fisher Scientific, Waltham, MA, USA). Protein samples were denatured at 100 °C for 20 min prior to SDS-PAGE gel electrophoresis. Next, 5–7 μg of protein per sample was loaded per lane in 4–12% polyacrylamide gel. After electrophoresis, the gel was transferred onto a polyvinylidene fluoride membrane (PVDF) under 90 mV current on ice for 1 h. After transference, the PVDF membrane was washed once in Tris-buffered saline with 1% Tween 20 (TBST) (Thermo Fisher Scientific, Waltham, MA, USA) for 5 min and subsequently blocked in 5% BSA for 1 h before primary antibody incubation. All primary antibodies were incubated at 4 °C overnight. After primary incubation, PVDF membranes were washed 3 times in TBST for 5 min before secondary antibody application. Secondary antibodies labeled with horseradish peroxidase (HRP, Cell Signaling Technology, Danvers, MA, USA) were used at a concentration of 1:500 and incubated at room temperature for 1 h. After secondary antibody incubation, the PVDF membranes were washed in 3 times in TBST for 5 min and imaged using HRP chemiluminescence detection (Azure Radiance Q) and imaged on the Azure Biosystems C600 imaging system (Azure Biosystems, Inc., Dublin, CA, USA). Western blot analysis was performed using previously described methods described in greater detail in ref. [31].

### 2.18. RT-qPCR

RNA was extracted using the Qiagen RNAeasy or Zymo QuickElute kit (QIAGEN, Hilden, Germany) (Zymo Research Corp., Irvine, CA, USA). RNA was converted to cDNA using the Superscript IV VILO reverse transcriptase mastermix (Thermo Fisher Scientific, Waltham, MA, USA). cDNA was amplified using primers obtained from IDT and validated against the NIH Primer bank. Quantitative PCR was performed using SYBER Green (Thermo Fisher Scientific, Waltham, MA, USA). All transcripts were normalized to GAPDH expression and run in triplicate with no-RT and no template controls.

### 2.19. Confocal Microscopy

Confocal images were acquired using a Leica TCS SP5 confocal microscope (Wetzlar, Germany). Z-stacks were captured using a stepwise interval of 1 µm. Images were captured at 20×, 40×, and 63× magnification and were further magnified using a zoom factor of 1.6 to obtain 100× images.

### 2.20. Image Analysis

All image analysis was performed in FIJI Image J (ImageJ2, version 2.16.0/1.54p; available at https://imagej.net/). Volume was calculated using manual tracing functions and measurement tools. Immunohistochemistry markers were quantified manually. Positivity was determined by pixel measurement and determining the pixel density of clearly positive markers in relation to non-specific background signal.

### 2.21. Statistical Analyses

All statistical analyses were performed using Graph Pad Prism 10 (GraphPad Prism version 10.4.1 (532) for macOS; GraphPad Software, San Diego, CA, USA; available at https://www.graphpad.com/). Two group analyses were performed using unpaired two-tailed *t*-tests. Variance was assessed using F tests. Three and four group analyses were assessed using one-way ANOVA with Tukey’s multiple comparison post hoc test.

## 3. Results

### 3.1. Noggin Is Transported to the Apical Membrane of Neurons

**Figure 1:**
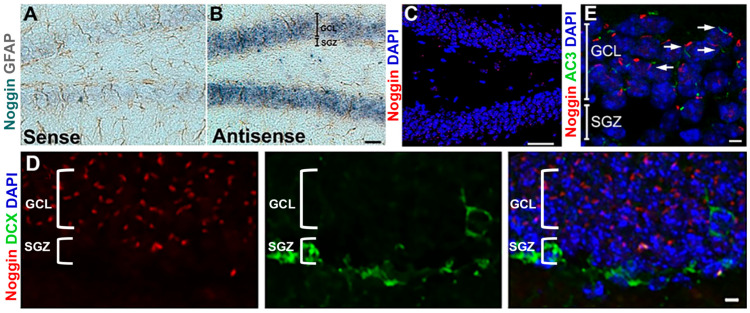
Noggin is expressed in the granule cell layer but not in the subgranular zone of the dentate gyrus. (**A**) Noggin mRNA in situ hybridization in the dentate gyrus of adult mice using a sense strand probe and (**B**) using an antisense strand probe. (**C**) Immunostaining for noggin (red) in the DG. (**D**) Immunostaining for noggin (red) and doublecortin (DCX, green), showing spatial separation of noggin- and DCX-expressing cells in the GCL and SGZ, respectively. (**E**) Immunostaining for the primary ciliary marker adenylate cyclase type III (green) and noggin (red) showing noggin associating near primary cilia (white arrows) in the GCL but not in the SGZ. Scale bars: (**A**–**C**): 100 μm, (**D**,**E**): 10 μm.

Despite the powerful antagonistic effect of noggin on BMP signaling in the neurogenic niche, the cellular localization of noggin is not fully understood. Using in situ hybridization, we confirmed noggin expression in DG granule neurons (Figure 1A,B), consistent with prior reports [23,24,25,26,32]. Immunohistochemistry demonstrated noggin protein expression in granule cell layer neurons (Figure 1C). Importantly, noggin immunoreactivity was not observed in the subgranular zone (SGZ). Conversely, doublecortin-stained cells were seen in the SGZ but not in the granule cell layer (Figure 1D). The spatial distribution of noggin within granule neurons was discrete, revealing an apical cylindrical pattern of noggin accumulation adjacent to primary cilia labeled with ciliary marker adenylyl cyclase type III (ACIII) (Figure 1E).

**Figure 2:**
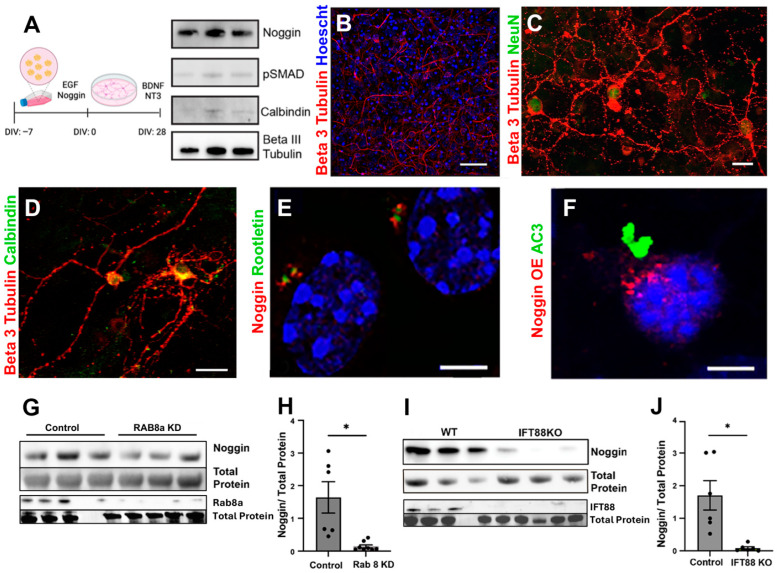
Noggin is apically targeted by RAB8a to the base of the primary cilium. (**A**) Schematic of in vitro culture timeline for granule neuron differentiation and maturation and immunoblot of neurons cultured 28 DIV showing noggin, pSMAD, and Calbindin expression. (**B**) Immunostaining of 28 days in vitro cultured neurons for beta III Tubulin (red) and Hoechst (blue). (**C**) Immunostaining of 28 DIV cultured neurons for beta III tubulin (red) and NeuN (green). (**D**) Immunostaining of 28 DIV cultured neurons for beta III tubulin (red) and calbindin (green). (**E**) Immunostaining of 28 DIV cultured neurons for noggin (red) and the ciliary associated protein rootletin (green), showing spatial proximity of noggin and rootletin. (**F**) Representative 28 DIV neuron transfected with noggin-OFP overexpression (red) and immunostained for adenylate cyclase type III (green) showing apical localization of noggin-OFP at the base of the primary cilium. (**G**) Representative western blot probed for noggin and RAB8a protein in WT and siRNA knockdown of RAB8a in cultured 28 DIV neurons. (**H**) Quantification analysis of immunoblot from the experiment in M; *n* = 6, * *p* < 0.05. (**I**) Representative western blot probed for noggin and IFT88 protein in WT and IFT88 KO in vitro. (**J**) Quantification analysis of immunoblot from the experiment in (**I**); *n* = 6, * *p* < 0.05. Scale bars: (**B**): 100 μm; (**C**,**D**): 10 μm; (**E**,**F**): 5 μm.

To further assess noggin internal trafficking, expression, and regulation, we utilized an in vitro model of hippocampus-derived neurons differentiated from postnatal neurosphere cultures. In brief, neural progenitor cells (NPCs) were harvested from P1–P3 postnatal mice, cultured as neurospheres, differentiated in the presence of BDNF and NT3, and allowed to mature for 28–32 DIV before examination (Figure 2A). At the time of collection, neurons expressed noggin, NeuN, and calbindin protein as assessed by western blot and ICC (Figure 2A–D). Some S100b+ astrocytes persisted in cultures but, importantly, progenitor cells (nestin+ or doublecortin+) were absent, cultures did not express Gli1, and there were no proliferating (BrdU+) cells. Immunocytochemical analysis of these cultures revealed that noggin protein was present only in neurons and not in astrocytes (Supplementary Figure S1A,B). In mature neurons, noggin was distributed in distinct apical puncta along with rootletin, a protein that specifically localizes to a membrane domain found at the base of cilia known as the ciliary “pocket region” (Figure 2E) [33]. To further assess these findings, we overexpressed an orange fluorescent protein (OFP)-tagged noggin fusion protein and again observed noggin associated with the base of AC3+ primary cilia (Figure 2F).

Apically targeted proteins are transported from the Golgi apparatus to the apical surface via the small GTP-binding vesicular transport protein RAB8a [34]. To determine the effects of apical targeting on noggin expression, we examined the effects of siRNA-mediated knockdown of RAB8a and found that noggin protein was reduced in knockdown cultures (*p* < 0.02) compared to vehicle-treated controls (Figure 2G,H). We next asked whether disrupting the primary ciliary structure and signaling affects noggin expression and protein localization. The intraflagellar transport (IFT) protein, IFT88, traffics both signaling proteins and the ciliary machinery necessary for structural integrity of the ciliary axoneme [35], and ablation of IFT88 leads to structural collapse of the primary cilia and loss of ciliary GPCR-dependent sensory function [36,37]. We generated neurosphere-derived neuronal cultures from IFT88^flx/flx^ animals followed by neuron-specific Cre-mediated ablation of IFT88 and observed reduced noggin expression (*p* < 0.02) in IFT88^−/−^ cultures compared to empty vector-treated controls (Figure 2I,J). Together, these results localized noggin to the apical membrane and suggested that noggin protein levels are dependent upon the vesicular trafficking protein, RAB8a, and the ciliary intraflagellar transport protein, IFT88.

### 3.2. Ciliary Sonic Hedgehog Signaling Regulates Noggin Expression and Release

**Figure 3:**
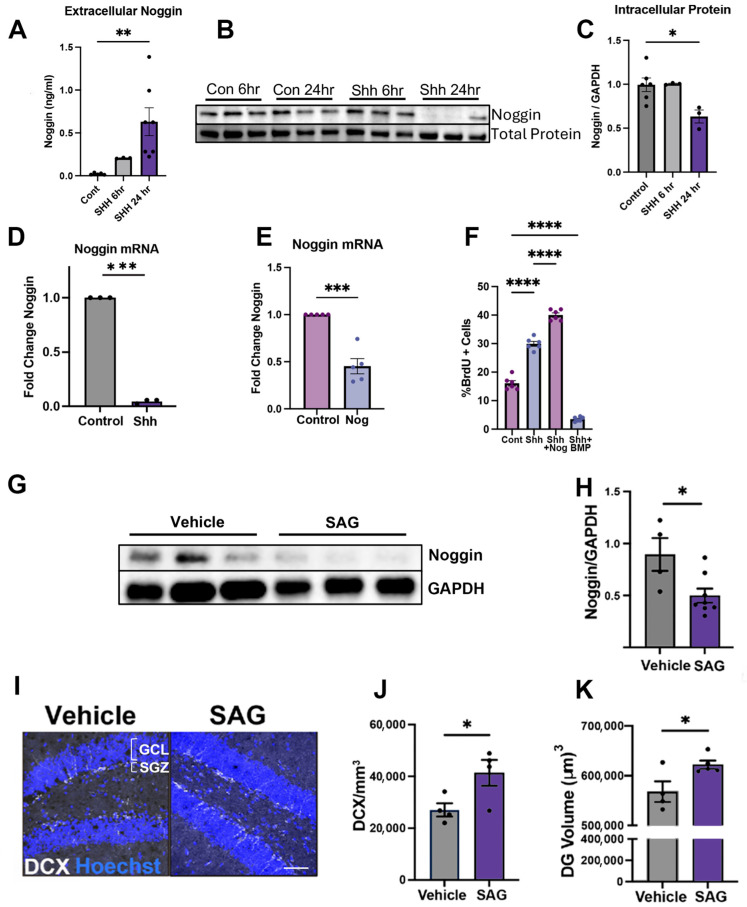
Ciliary sonic hedgehog signaling modulates noggin expression and release. (**A**) Quantification of extracellular noggin in the media of cultured neurons following Shh treatment for 6 and 24 h as assessed by ELISA; *n* = 5, ** *p* < 0.01. (**B**) Representative western blot probed for noggin protein in neurons cultured 28 DIV treated with Shh for 6, 24 h. (**C**) Quantification of intracellular noggin in cultured cells treated for 6 and 24 h with Shh; *n* = 3, * *p* < 0.05. (**D**) Noggin mRNA expression levels in cultured neurons treated with Shh for 24 h; *n* = 3, *** *p* < 0.001. (**E**) Noggin mRNA expression levels in cultured neurons treated with 250 ng/mL noggin for 24 h; *n* = 5, *** *p* < 0.001. (**F**) Quantification of % BrdU-positive NPC cultured in the presence of FGF and BrdU containing control medium, Shh, Shh + noggin, or Shh + BMP, showing the additive effect of noggin on Shh induced proliferation and a significant downregulation of proliferation when BMP signaling is unopposed by noggin; *n* = 6, **** *p* < 0.0001. (**G**) In vivo representative western blot probed for noggin in both control and SAG treated animals. (**H**) Quantification of western blot probed for intracellular noggin in control and SAG treated animals as seen in (**H**); *n* = 6, * *p* < 0.05. (**I**) Representative image of immunohistochemistry of the dentate gyrus stained for doublecortin (DCX) in both control and SAG-treated adult animals. (**J**) Quantification of DCX from experiment represented in (**J**); *n* = 4, * *p* < 0.05. (**K**) Quantification of DG volume in control and SAG-treated animals from experiment represented in (**J**); *n* = 4, * *p* < 0.05. Data shown as mean ± SEM. Multiple comparisons test with one-way ANOVA for multiple group experiments and unpaired two-tailed Student’s *t* test for two group experiments. * *p* < 0.05; ** *p* < 0.01; *** *p* < 0.001; **** *p* < 0.0001. Scale bar: (**I**): 100 μm.

Our observations that noggin expression is altered by disruption of the ciliary structure led us to ask whether signaling specifically through cilia regulates noggin expression and release. Sonic hedgehog signaling is mediated through primary cilia and functions in opposing gradients to BMP during the development of the central nervous system [29,38]. Therefore, we hypothesized that Shh signaling may regulate noggin expression and release in our cell culture model. Shh treatment of neurosphere-derived neurons resulted in a substantial ~30-fold increase (*p* < 0.01) in extracellular noggin levels compared to vehicle-treated controls (Figure 3A) and a significant reduction of noggin protein levels detected in cell culture lysate (*p* < 0.05) (Figure 3B,C). Examination of noggin mRNA after Shh exposure revealed a significant reduction of noggin mRNA after 24 h of Shh treatment, (*p* < 0.001) (Figure 3D). These data suggested that downregulation of noggin mRNA expression could be due to feedback inhibition from extracellular noggin signaling. To test this hypothesis, we added noggin to the media of neurosphere-derived cultures for 24 h and found that treatment resulted in a significant >50% decrease in noggin mRNA transcripts (*p* < 0.001) (Figure 3E).

Noggin and Shh overlap in neurogenic function, so we next examined whether Shh and noggin exerted synergistic effects in the neurogenic niche modeled by progenitor cell cultures. Progenitor cells were derived from neurospheres, dissociated into single cells, and cultured. We treated progenitor cell cultures with Shh and measured BrdU incorporation alone and in the presence or absence of noggin and BMP. Shh treatment resulted in a ~2-fold increase in the number of cells labeled by BrdU, as anticipated (Figure 3F). Co-treatment with both Shh and noggin resulted in an approximate 3-fold increase in BrdU incorporation compared to vehicle-treated controls (*p* < 0.0001). However, co-treatment with Shh and BMP significantly reduced the percentage of proliferating cells to levels below that observed in vehicle-treated controls (Figure 3F). This suggested that noggin acts synergistically with Shh to increase neurogenesis by counteracting BMP signaling.

We extended our findings in vivo, modulating Shh signaling with Smoothened agonist (SAG) in adult mice. SAG treatment, administered intraperitoneally for 5 days, resulted in a significant reduction in noggin expression (*p* < 0.05) (Figure 3G,H), consistent with the extraordinary release we observed in vitro. Treatment with SAG resulted in an increase in the number of doublecortin-positive newborn neurons and volume of the dentate gyrus (*p* < 0.05) (Figure 3I–K).

### 3.3. Ciliary Somatostatin Signaling Regulates Noggin Dynamics

**Figure 4:**
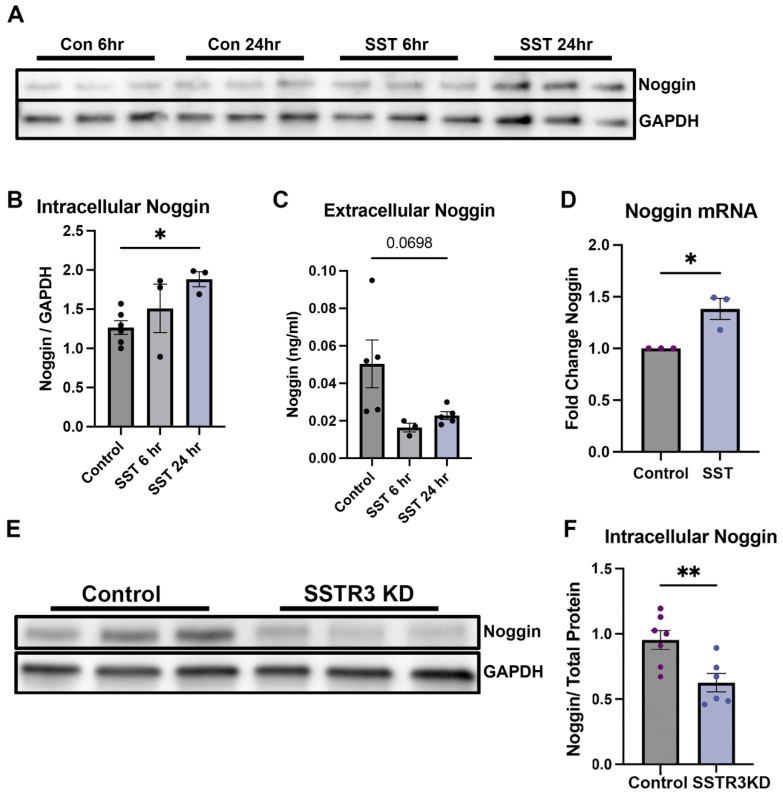
Ciliary somatostatin signaling modulates noggin expression and release. (**A**) Representative western blot probed for noggin following treatment of 28 days in vitro cultured cells with SST for 6 and 24 h. (**B**) Quantification of western blot probed for noggin following treatment of 28 DIV cultured cells with SST for 6, 24 h; *n* = 6, * *p* < 0.05. (**C**) Quantification of extracellular noggin in the media of cultured neurons following SST treatment for 6 and 24 h as assessed by ELISA; *n* = 5, *p* = 0.0698. (**D**) Noggin mRNA expression levels in cultured neurons treated with SST for 24 h; *n* = 3, * *p* < 0.05. (**E**) Representative western blot probed for noggin with siRNA knockdown of SSTR3 in 28 DIV cultured neurons. (**F**) Quantification of western blot probed for noggin showing levels of intracellular noggin following SSTR3 KD in vitro; *n* = 6, ** *p* < 0.01. Data shown as mean ± SEM. Multiple comparisons test with one-way ANOVA for multiple group experiments and unpaired two-tailed Student’s t test for two group experiments. * *p* < 0.05; ** *p* < 0.01.

Our observation that ciliary Shh signaling significantly modulated noggin dynamics prompted us to study other ciliary GPCR signaling pathways. Because SSTR3 is localized exclusively to primary cilia, the study of somatostatin signaling pathways became of considerable interest. SST is a neurotransmitter expressed by GABAergic interneurons in the hippocampus and exerts an inhibitory effect on granule neurons [39]. We treated neurosphere-derived neuron cultures with SST for 24 h and observed significantly higher cellular noggin levels after treatment (*p* < 0.05) (Figure 4A,B). Treatment with SST in vitro for 6 and 24 h showed a trend toward reduced extracellular noggin levels compared to baseline (*p* = 0.0698) (Figure 4C). Assessment of noggin mRNA after 24 h of SST treatment showed higher expression of the noggin transcript, likely due to the lack of noggin feedback inhibition on noggin transcription due to lowered noggin release (*p* < 0.05) (Figure 4D). SST acts through 5 unique receptor subtypes; however, the SSTR3 receptor is expressed only in cilia [40]. To target SST signaling specifically within primary cilia, we transfected cultured granule neurons with an siRNA against SSTR3. Knockdown cultures had significantly lower levels of noggin protein compared to vehicle controls (*p* < 0.01; Figure 3E,F). Together, these results supported the conclusion that neuronal activity regulates dentate granule neuron noggin expression and release by cilia.

### 3.4. Neuronal Activity Stimulates Noggin Expression and Release

**Figure 5:**
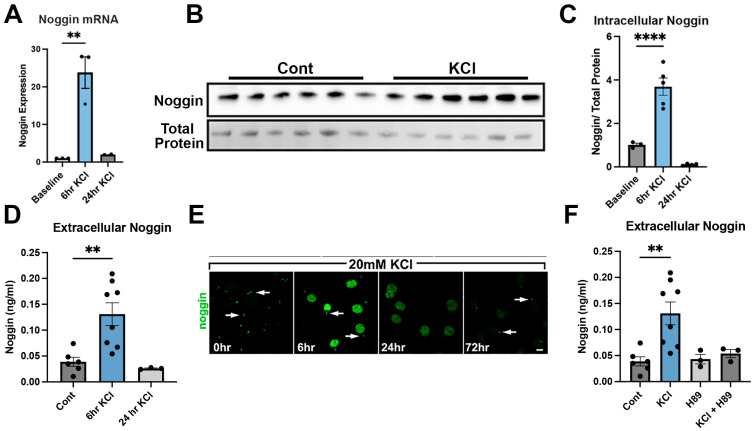
Mature neuron activity increases noggin expression and release. (**A**) Noggin mRNA expression levels in neurons cultured 28 days in vitro treated with 20 mM KCl collected at 6 h and 24 h; *n* = 3, ** *p* < 0.01. (**B**) Representative western blot probed for noggin protein in neurons cultured 28 DIV treated with 20 mM KCl for 6 h. (**C**) Quantification of western blot probed for noggin showing levels of intracellular noggin after cultured cells were treated with 20 mM KCl for 6 h and 24 h; *n* = 4, **** *p* < 0.0001. (**D**) Quantification of extracellular noggin in the media of cultured neurons following 20 mM KCl treatment for 6 and 24 h as assessed by ELISA; *n* = 4, ** *p* < 0.01. (**E**) Representative immunocytochemistry showing 28 DIV neurons treated with 20 mM KCl and stained for noggin (green) showing ciliary distribution of noggin staining (indicated by white arrows) at 0 h of treatment, increased ciliary and cytoplasmic noggin expression at 6 h, loss of ciliary noggin distribution and diminished cytoplasmic expression of noggin at 24 h, and return to baseline ciliary distribution of noggin at 72 h. (**F**) Quantification of extracellular noggin in the media of cultured neurons following 20 mM KCl 6 h treatment. Addition of the PKA inhibitor, H89, blocks the effect of KCl depolarization on noggin release; *n* = 6, ** *p* < 0.01 Data shown as mean ± SEM. Multiple comparisons test with one-way ANOVA for multiple group experiments. ** *p* < 0.01; **** *p* < 0.0001. Scale bars: (**E**): 5 μm.

Given the known inhibitory effects of SST signaling on neurons and our previous finding that enhanced activity in the neurogenic niche increased noggin expression [41], we examined the effects of neuronal depolarization on noggin dynamics. To determine whether neuronal activity modulates expression and release of noggin in a cell autonomous manner, we depolarized cultured neurons with 20 mM KCl for 6, 24, and 72 h and examined noggin mRNA and protein expression and noggin release (Figure 5). At 6 h, we observed greater noggin mRNA expression compared to baseline (*p* < 0.01) (Figure 5A). Noggin expression returned to baseline at the 24 h timepoint (Figure 5A). Similarly, noggin protein levels were significantly higher after 6 h of KCl treatment (*p* < 0.0001) (Figure 5B,C). Protein levels of noggin were reduced at 24 h, trending to levels below that of baseline cultures (Figure 5C). Extracellular noggin was measured via sandwich ELISA and revealed significant extracellular release at 6 h, with a decline in measurable noggin in media detected at 24 h (*p* < 0.01) (Figure 5D). Noggin immunocytochemistry confirmed a change in noggin localization with KCl treatment (Figure 5E). At baseline, noggin immunoreactivity was localized proximal to the cell membrane in a discrete barrel structure. After 6 h of KCl depolarization, noggin immunoreactivity increased throughout the cytoplasm. At 24 h of KCl depolarization, noggin staining was greatly diminished throughout the cytoplasm. At 72 h, barrel-shaped puncta were again observed with low signal distribution throughout the cytoplasm (Figure 5E), suggesting temporally dynamic changes in the localization of noggin within the cell. Protein kinase A (PKA) mediates the signaling effects of membrane depolarization [38]. Treatment of cultured neurons with the PKA inhibitor H89 [39] blocked the KCl-mediated increase in noggin expression (Figure 5F). H89 alone had no effect on noggin protein level. These findings suggested that PKA signaling, which can be primary cilium mediated [42,43], is necessary for membrane depolarization-induced noggin secretion from cultured neurons.

**Figure 6:**
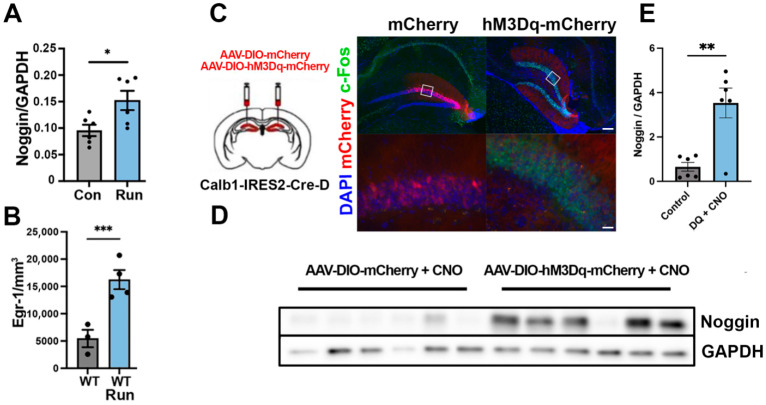
Chronic activation of mature granule cells upregulates noggin expression. (**A**) Quantification of western blot probed for noggin in the DG of either control animals or animals housed with a running wheel for 2 weeks; *n* = 6 per group, * *p* < 0.05. (**B**) Quantification of immunohistochemistry of DG stained for Egr-1 in WT animals housed both without and without running wheel access for 2 weeks; *n* = 3, *** *p* < 0.001 compared to control group. (**C**) Experimental scheme and representative images of bilateral stereotactic injections of adeno-associated virus (AAV) carrying Cre-dependent expression of either control (AAV-DIO-HM3Dq-mCherry) (left column) or excitatory DREADD receptor hM3Dq (AAV-DIO-HM3Dq-mCherry) (right column) into the dentate gyrus of Calb1-Cre mice and treated with CNO 0.25 mg mL^−1^ ad libitum in drinking water for 2 weeks with immunostaining for DAPI (blue), mCherry (red), and c-Fos (green). (**D**) Western blot analysis of hippocampal tissue from animals represented in (**C**), probed for noggin. (**E**) Quantification of the data represented in (**D**); *n* = 6, ** *p* < 0.01. Data shown as mean ± SEM. Multiple comparisons test with one-way ANOVA for multiple group experiments and unpaired two-tailed Student’s *t* test for two group experiments. * *p* < 0.05; ** *p* < 0.01; *** *p* < 0.001. Scale bars: (**C**, top panels): 300 μm; (**C**, bottom panels): 20 μm.

Consistent with previous reports by our group and others, we found that mice exposed to running had greater noggin mRNA expression and protein levels in the DG (*p* < 0.05) (Figure 6A) [19]. Running also led to an increase in immediate early gene (IEG) expression, such as Egr-1, in dentate granule neurons (*p* < 0.001) (Figure 6B), consistent with an increase in neuronal activity [44]. To determine whether increases in neuronal activity specifically in calbindin-expressing mature granule cells might modulate noggin dynamics, we used Calb1-Cre mice to selectively activate this noggin-expressing population of neurons using bilateral stereotactic injections of adeno-associated virus (AAV) carrying Cre-dependent expression of excitatory DREADD receptor hM3Dq (AAV-DIO-HM3Dq-mCherry) into the dentate gyrus (DG) (Figure 6C). Calbindin-expressing neuron activation was maintained by administering Clozapine-N-Oxide in drinking water ad libitum over a two-week period. Immunohistochemistry confirmed hM3Dq-induced neuronal activation, as evidenced by elevated c-Fos expression in Calbindin-expressing, mCherry-positive mature granule cells (Figure 6C). Western blot analyses revealed significant increases in noggin protein levels in the DG of hM3Dq-expressing animals compared to mCherry-expressing controls (*p* < 0.01) (Figure 6D,E), consistent with our in vitro findings that neuronal activity enhanced noggin expression.

## 4. Discussion

Signaling through primary cilia facilitates neuronal function in both the developing and adult nervous systems [27,45,46], and defective ciliary function leads to both psychiatric and neurodegenerative disorders [40,47]. Cilia receive and integrate the effects of many extracellular signals, including Shh, somatostatin, and others [13,29,48]. Our findings indicate that cilia also play a role in extracellular signaling by regulating the release of noggin. Ciliary release of noggin is increased by nerve impulse activity and by sonic hedgehog signaling, and it is decreased by inhibitory input mediated by ciliary somatostatin receptors.

During development, Shh and BMPs have opposing gradients that work to pattern the CNS along the dorsoventral axis [38]. In adults, Shh signaling enhances neurogenesis and regulates hippocampus-dependent behavior, while BMPs negatively regulate the birth of new neurons and are associated with worsened hippocampus-dependent behavioral outcomes [28,29,44,49]. Noggin is a BMP inhibitor in the hippocampus that is upregulated by physiologic stimuli such as running and environmental enrichment, and these manipulations result in increased levels of hippocampal neurogenesis [3,19,20,41]. However, the cellular expression of noggin in the hippocampus and the mechanisms governing its release are not well known. In this study, we determined that in the adult hippocampus, noggin is apically localized in DG granule neurons in association with cilia. We confirmed our immunohistochemical results using several different noggin antibodies both in vivo and in vitro using neurosphere-derived cultures. To further confirm the findings, we overexpressed an OFP-tagged noggin construct in C57B/6 neurosphere-derived cultures and found that it localized intracellularly to the apical membrane adjacent to the ciliary marker adenylate cyclase III (Figure 2F). Finally, we found that disruption of apical targeting by knockdown of RAB8a and that disruption of cilia by knockout of IFT88 each reduced neuronal noggin expression. Our observations thus clearly localize noggin to the apical membrane of DG granule neurons and demonstrate that noggin protein levels are dependent upon RAB8a and IFT88. Apical targeting of noggin has been described in epiblasts where noggin is apically targeted for secretion, while BMP4 is basally targeted and secreted [50]. BMP receptors have similarly been localized to the apical cell membrane in some peripheral cell types [51]. Our findings of the apical localization of noggin within granule neurons thus fits within the larger landscape of previous work with non-neuronal cells.

Noggin functions extracellularly by competing with membrane receptors for BMP ligands and therefore requires extracellular release. We found that neuronal activation induced via KCl depolarization in vitro or chemogenetic activation in vivo led to noggin release, while treatment with the PKA inhibitor H89 and inhibitory neurotransmitter SST prevented noggin release. Ciliary cAMP activates PKA during neuronal activation and leads to downstream activation of various G protein-coupled receptors [52]. We observed greater noggin levels in exercised animals compared to non-exercised controls, with significantly greater Egr-1 expression, which is also activated by the cAMP/PKA/CREB signaling pathway [53]. Pharmacologic and chemogenetic studies reveal that hippocampal activity is sufficient to modulate BMP signaling, Shh expression, noggin expression, and expression of immediate early genes [19,41,54,55]. Importantly, we observed that a reduction in cilium-specific SSTR3 levels led to lower noggin expression, similar to that observed in cells with impaired ciliary structures as the result of IFT88 deletion or with RAB8a knockdown. Taken together, these findings suggest that primary cilia play a crucial role in activity-dependent release of noggin from the cell.

Shh treatment of cultured hippocampal neurons resulted in rapid and profound extracellular release of noggin. Because Shh signaling is cilium mediated, this directly implicates cilia in the control of noggin release. The neuronal cultures were completely devoid of *gli* expression, indicating the involvement of non-canonical Shh signaling in regulating noggin release. The cellular source (s) of Shh in the hippocampus is (are) not fully characterized, although dentate gyrus mossy fibers appear to be one source [56]. Mossy cells provide excitatory input to dentate granule neurons, consistent with our observation that excitatory input promotes noggin release. Importantly, mossy cells also innervate inhibitory interneurons, including the SST interneuron population [57], consistent with the role of mossy cells in modulating excitatory/inhibitory tone in the DG [57,58]. We found that SST reduced noggin release and increased cellular levels of noggin, while knockdown of the cilium-specific SSTR3 receptor reduced levels of noggin (Figure 3). These results fit within the larger context of recent work demonstrating that BMP signaling modulates the excitatory and inhibitory balance of cortical neurons [59]. In addition, ciliary signaling has also been shown to regulate the excitatory–inhibitory tone of postnatal pyramidal neurons [27]. Taken together, our findings suggest that neuronal activity-regulated noggin release may provide feedback BMP signaling inhibition, leading to modulation of the excitatory–inhibitory balance of the DG circuitry.

We observed a profound stimulatory effect of Shh on noggin extracellular release and reduced cellular levels of noggin mRNA and protein in vitro and in vivo. The reduction in noggin mRNA expression with Shh treatment suggests a regulatory feedback mechanism that may explain lower expression of noggin transcript measured at the 24 h time point where we detected significant noggin release. This is consistent with prior observations in both the developing nervous system and in other organs with feedback mechanisms [16,60,61,62]. Because Shh and noggin have overlapping functions and similarly increase hippocampal neurogenesis, further work needs to be done to understand the synergistic and partially overlapping effects of these two morphogens and the extent of potential signaling crosstalk. Importantly, the source of Shh in the adult hippocampus remains incompletely defined. Recent studies identify hilar mossy cells, medial septal projections, and local interneurons as potential neuronal sources, with transcriptomic and in situ evidence supporting neuron-specific Shh expression under baseline conditions [63,64,65]. Future work could selectively alter the activation of these circuits to investigate potential signaling crosstalk between Shh and Noggin in vivo.

Furthermore, these mechanistic findings may have functional implications in the context of hippocampus-dependent cognition across various states of health and disease. Increased noggin expression and reduced BMP signaling have been linked to improved cognitive outcomes, both during normal aging and in pathological conditions. Notably, recent work has shown that noggin overexpression limits tau phosphorylation in human iPSC-derived APOE4 models and, in vivo, rescues phospho-tau deposition and hippocampus-dependent memory in P301S mice [66], suggesting that noggin levels may influence cognition and phospho-tau levels in Alzheimer’s disease and other tauopathies. Our results further suggest that granule cells and neuronal primary cilia contribute to the regulation of noggin signaling, offering new insight into how neuron-intrinsic mechanisms may shape the functional output of the neurogenic niche.

This study is not without limitations. Neurosphere-derived granule neuron cultures are largely neuronal, but a small percentage of glial cells remain. Importantly, there were no detectable progenitor cells in the cultures, and there was no detectable noggin expression in the glia. Nevertheless, we cannot rule out the possibility that the presence of glia influenced neuronal phenotypes, specifically in experiments with treatment of exogenous factors including KCl, H89, or Shh. In addition, we focused on a 24 h time point to capture the early regulatory effects of Shh and noggin while minimizing confounding changes associated with prolonged treatment in vitro. While this approach provided a clear view of the initial feedback, it also highlights the need to examine the temporal dynamics of this regulation in vivo. Such studies would be further strengthened by targeting specific Shh-producing circuits, as identified above, to better understand how sustained or context-dependent signaling influences noggin expression and neurogenic outcomes. Finally, our findings support a model in which Shh regulates noggin release through a non-canonical signaling mechanism, characterized by the absence of Gli1 expression, dependence on primary cilia, and involvement of the GPCR SSTR3. This aligns with established models of Gli-independent Shh signaling in postmitotic neurons, where smoothened activates intracellular pathways without inducing transcriptional responses. Non-canonical Shh signaling has also been shown to engage PI3K activity in various contexts, further supporting the potential for transcription-independent regulation [67,68]. While we did not experimentally assess the full complement of Gli family transcription factors or downstream signaling intermediates, our findings align strongly with established non-canonical mechanisms. These results provide a framework for future studies aimed at defining how neuron-intrinsic, cilia-dependent Shh signaling drives noggin release and shapes BMP pathway dynamics in the adult hippocampus.

Overall, the results of this study expand our current understanding of primary cilia in the context of hippocampal neurogenesis, BMP signaling, and the role of primary cilia on hippocampal-dependent behavior and cognition. Our results demonstrate that noggin is expressed and localized to the mature granule cell population. Further, this work reveals that primary cilia regulate both the expression and release of noggin in the DG. In the larger context of brain physiology, the results of this study suggest that ciliary signaling may play a functional role in mediating cognitive and affective behavior through the regulation of BMP antagonism. More generally, while primary cilia are generally viewed as sensory organelles that transduce chemical and mechanical stimuli from the environment, our observations indicate that they also have an extracellular signaling role.

## Data Availability

Original data created for the study will be available in a persistent OSF repository upon publication.

## Supplementary Materials

The following supporting information can be downloaded at: https://www.mdpi.com/article/10.3390/cells14201607/s1, Figure S1: Neuronal identity and Noggin localization in mature hippocampal cultures. (**A**) Quantification of neuronal (NeuN^+^) and astrocytic (GFAP^+^) populations in neurosphere-derived hippocampal cultures maintained for 28–32 days in vitro both with and without 1 µM AraC to limit glial proliferation. Data represent mean ± SEM from *n* = 8 cultures. (**B**) Representative immunofluorescence images from 28 DIV mature cultures showing neurons transfected with Noggin-OFP (red). Noggin signal colocalizes with MAP2^+^ neurons (magenta; white arrows) and is absent from MAP2^−^ cells (orange arrows), indicating neuron-specific localization. AC3^+^ primary cilia are labeled in green.
