# Supplementary material for: Neuronal Primary Cilia Mediate Noggin Release to Enable Extracellular Signaling
Source: Cells. 2025 Oct 16;14(20):1607. doi: 10.3390/cells14201607 (PMC12564481; doi:10.3390/cells14201607)
Supplement: Supplementary file 1 [file cells-14-01607-s001.zip › cells-3792848-supplementary.pdf]

Supplementary Materials

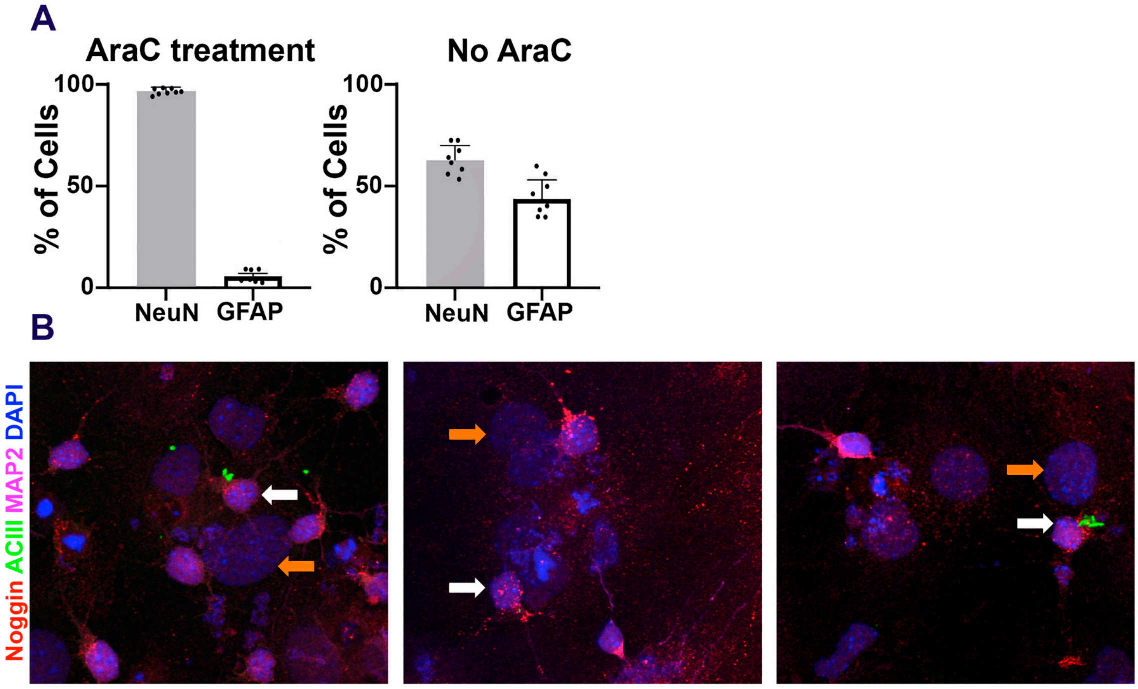

Figure S1. Neuronal identity and Noggin localization in mature hippocampal cultures. (**A**) Quantification of neuronal (NeuN$^+$) and astrocytic (GFAP$^+$) populations in neurosphere-derived hippocampal cultures maintained for 28–32 days in vitro both with and without 1 μM AraC to limit glial proliferation. Data represent mean ± SEM from n = 8 cultures. (**B**) Representative immunofluorescence images from 28 DIV mature cultures showing neurons transfected with Noggin-OFP (red). Noggin signal colocalizes with MAP2$^+$ neurons (magenta; white arrows) and is absent from MAP2$^-$ cells (orange arrows), indicating neuron-specific localization. AC3$^+$ primary cilia are labeled in green.
